# Revolutionizing dermatology: harnessing mesenchymal stem/stromal cells and exosomes in 3D platform for skin regeneration

**DOI:** 10.1007/s00403-024-03055-4

**Published:** 2024-05-25

**Authors:** Mesude Bicer

**Affiliations:** Department of Bioengineering, Faculty of Life and Natural Sciences, Abdullah Gul University, Kayseri, 38080 Turkey

**Keywords:** Mesenchymal stem/Stromal cells, Exosomes, 3D biomaterials, Skin diseases, Skin regeneration

## Abstract

Contemporary trends reveal an escalating interest in regenerative medicine-based interventions for addressing refractory skin defects. Conventional wound healing treatments, characterized by high costs and limited efficacy, necessitate a more efficient therapeutic paradigm to alleviate the economic and psychological burdens associated with chronic wounds. Mesenchymal stem/stromal cells (MSCs) constitute cell-based therapies, whereas cell-free approaches predominantly involve the utilization of MSC-derived extracellular vesicles or exosomes, both purportedly safe and effective. Exploiting the impact of MSCs by paracrine signaling, exosomes have emerged as a novel avenue capable of positively impacting wound healing and skin regeneration. MSC-exosomes confer several advantages, including the facilitation of angiogenesis, augmentation of cell proliferation, elevation of collagen production, and enhancement of tissue regenerative capacity. Despite these merits, challenges persist in clinical applications due to issues such as poor targeting and facile removal of MSC-derived exosomes from skin wounds. Addressing these concerns, a three-dimensional (3D) platform has been implemented to emend exosomes, allowing for elevated levels, and constructing more stable granules possessing distinct therapeutic capabilities. Incorporating biomaterials to encapsulate MSC-exosomes emerges as a favorable approach, concentrating doses, achieving intended therapeutic effectiveness, and ensuring continual release. While the therapeutic potential of MSC-exosomes in skin repair is broadly recognized, their application with 3D biomaterial scenarios remains underexplored. This review synthesizes the therapeutic purposes of MSCs and exosomes in 3D for the skin restoration, underscoring their promising role in diverse dermatological conditions. Further research may establish MSCs and their exosomes in 3D as a viable therapeutic option for various skin conditions.

## Introduction

Skin injuries present a substantial and multifaceted challenge within contemporary clinical practice, imposing a significant financial burden on both patients and the healthcare system [1]. Furthermore, chronic wounds give rise to pronounced patient morbidity, exerting detrimental effects on their quality of life, while concurrently exacerbating sensations of pain, stress and depression [2]. Currently, the predominant therapeutic approach employed to expedite wound healing involves the application of biological agents, such as growth factors and cytokines [3, 4]. Nonetheless, these therapeutic modalities are predominantly characterized by their costliness, protracted durations of treatment, and inefficacy due to their limited clinical efficacy and safety profile. It is also worth noting that over half of chronic wounds exhibit significant resilience to standard treatments, and these approaches inherently lack the capacity to rectify scarring concerns [5, 6].

Contemporary trends underscore a burgeoning interest in the utilization of interventions rooted in regenerative medicine for skin rejuvenation and skin regeneration, encompassing a wide spectrum of applications, such as trauma, burns, cutaneous wounds, and dermatological maladies [7]. Remarkably, mesenchymal stem/stromal cells (MSCs) and their secreted extracellular vesicles, notably exosomes, have surfaced as promising entities for the revitalization of compromised tissues, both within the realms of preclinical investigations and clinical trials [8, 9]. Recent advancements MSC-based therapeutic research have unveiled the potential of MSC-derived extracellular vesicles (EVs), particularly exosomes, in the context of cutaneous wound healing and skin regeneration. Innovative approaches have been devised to strengthen the regenerative properties of MSCs and their exosomes, often involving their incorporation into 3D biomaterial scaffolds. The integration of MSC-exosomes into 3D platforms has introduced a ground-breaking, cell-free therapeutic paradigm, eliciting significant enthusiasm within clinical practice. Preclinical and clinical assessments involving chronic wound models have revealed encouraging outcomes, showcasing that engineered MSCs and their exosomes possess the capacity to stimulate angiogenesis, re-epithelization, granulation tissue formation, and mitigate inflammatory responses [10–13]. Furthermore, MSC-exosome-based therapies have exhibited heightened therapeutic efficacy, exerting beneficial effects across all stages of wound healing [14]. However, the application of MSC based exosome-based therapy in clinical setting may confront certain challenges. The intricate procedures involved in isolating, purifying, and scaling up the generation of exosomes constitute a significant impediment to transitioning exosome therapy from experimental settings to clinical application. The absence of standardized methodologies for exosome isolation results in variations in safety and quality features among exosome products. Ensuring consistency and safety considerations, the producibility of MSC-exosomes as a clinical product might be accepted in terms of pharmaceutical preparations. The application of bioengineering technology can be considered to modify exosome phenotypes, enabling the introduction of specific biological molecules carried by exosomes to enhance therapeutic efficacy or minimize undesirable effects [15–17]. Additionally, the integrity of biomaterials into MSC-exosomes-based interventions holds promise in addressing these practical impediments, potentially conferring systemic effects on wound closure, thereby enhancing their efficiency, and conferring supplementary therapeutic benefits [18]. Despite the absence of comprehensive empirical evidence, the modification of MSC-exosomes within 3D platforms is progressively gaining recognition as a valuable strategy to mitigate the limitations associated with clinical applications of naturally derived exosomes for the restoration of skin injuries.

This thorough review emphasizes the utilization of MSCs and their exosomes within 3D scaffold to augment their therapeutic potential in the context of healing of wound and regeneration of the skin. The integrity of wound dressings with biomaterials containing exosomes derived from MSCs stands as a promising approach, with the potential to enhance dosage concentration, engender desired therapeutic efficacy, and sustain the release of therapeutic agents over time. While the positive influence exosomes derived from MSCs on the process of wound healing has gained widespread recognition, the full potential of bioengineered, modified MSC-exosomes within a 3D framework has yet to be comprehensively elucidated. Our objective is to furnish a valuable overview that encompasses the intricate biological mechanisms modulated by MSCs and their exosomes when embedded within a 3D platform, all in service of supporting skin regeneration and wound amelioration. Additionally, we aim to expound upon the prospective applications of these modified MSC-exosomes within the realm of clinical practice.

## The complexity phases of cutanous wound healing: from hemostasis to inflammation

The healing of cutaneous wounds represents a dynamic physiological phenomenon that initiates in response to the disruption of skin integrity, involving the coordinated participation of various cellular entities along with their secreted biomolecules [19]. This intricate mechanism serves as an inherent protective response to the skin, serving to alleviate harm, thwart infections, and recover the anatomical integrity and functionality of the affected area [20]. The conventional sequence of healing for cutaneous wounds can be encapsulated within a series of interconnected stages: the Hemostatic stage, the Inflammation stage, the Proliferation stage and the Maturation stage, the latter of which is also referred to as the Remodeling stage [21]. Following skin injury, hemostasis, constituting the initial phase of the healing process, promptly instigates to arrest bleeding. Platelet activation through their interaction with collagen ultimately leads to platelet aggregation, resulting in the formation of stable clots catalyzed by thrombin, thereby playing a substantial role in the formation of a fibrin mesh and overall coagulation [22, 23]. Subsequently, the Inflammation phase, constituting the second step of wound healing, centers on clearing away microbial agents and residual matter, thereby establishing an environment conducive to the subsequent formation of new tissue [24, 25]. Key immunological actors, such as neutrophils and macrophages, assume pivotal roles in this phase, modulating not only bleeding but also countering infection through direct functionality and the secretion of an array of soluble mediators [26]. Recent scientific findings have unveiled the presence of two distinct anti-functional phenotypes among macrophages: the M1 phenotype, related to pro-inflammatory responses, and the M2 phenotype is linked to anti-inflammatory processes. After tissue damage, M1-type immune cells instigate pro-inflammatory activities essential for protective inflammatory responses and the clearance of injured tissues, while M2-type immune cells engender anti-inflammatory responses that support tissue regeneration [27]. Nevertheless, an excessive pro-inflammatory response and an insufficient anti-inflammatory response, can pose a risk of chronic wound development. The Proliferation phase of wound healing encompasses four essential regenerative events: wound filling through fibroblast proliferation, wound margin contraction facilitated by myofibroblast function through the production of extracellular matrix (ECM) components, and wound coverage [28]. Simultaneously, it becomes crucial to establish a novel network that supplies nutrients and oxygen to the burgeoning granulation tissue, thereby facilitating the process of angiogenesis. The Maturation or Remodeling phase involves the gradual alleviation of the inflammatory stage, the accumulation of collagen fibers, full coverage of the injured site by newly formed tissues [29, 30].

**Fig. 1:**
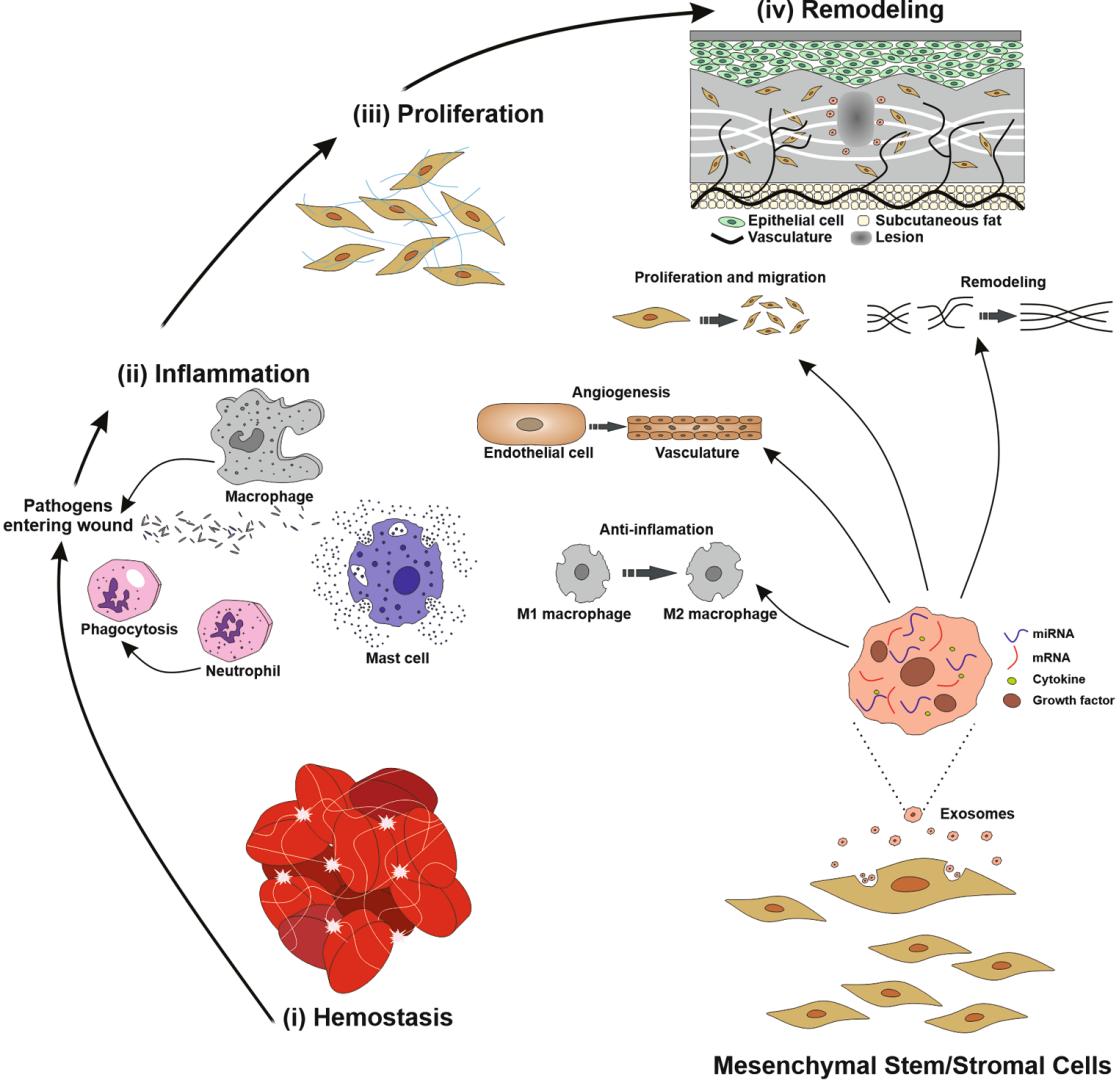
Processes that underlie the therapeutic impacts of MSCs and their exosomes concerning the recovery of wounds and the renewal of skin. The intricate process of skin wound healing is delineated, encompassing pivotal stages such as hemostasis through blood coagulation, the inflammatory response, cell proliferation, and tissue remodeling. Notably, MSCs and their extracellular vesicles, namely exosomes, exert a substantial influence on these stages. Their regulatory impact manifests in the diminution of tissue inflammation, the attenuation of the immune response, the facilitation of augmented cell migration and the encouragement of angiogenesis, and the orchestration of tissue remodeling

Crucially, the primary molecular orchestrators of the healing a wound encompass a cadre of proteins, inclusive of cytokines, chemokines, and growth factors [31, 32]. In recent decades, owing to the progress within the realm of regenerative medicine, substantial endeavors have been invested in exploring strategies to enhance tissue regeneration, thereby rectifying physiological deficits. These endeavors encompass the utilization of MSCs to potentiate a more efficacious healing response [33, 34]. Notably, the application of MSCs and their products, including exosomes and secretome, offers certain advantages in comparison of whole MSCs, offering a diminished risk of tumor formation and reduced susceptibility to immune rejection [35], a trend that is steadily on the rise. Indications propose that exosomes originating from MSCs have the capacity to encourage M2 polarization through the transfer of microRNAs (miRNAs). A variety of studies have corroborated that MSCs, and their exosomes possess therapeutic attributes akin to their parental cells, such as angiogenic potential and immune modulatory effects [35, 36]. For instance, He et al. documented that exosomes originating from bone marrow MSCs prompted the macrophage alignment with the M2 phenotype; with miR-223 derived from MSC exosomes implicated in the regulation of this polarization [37]. In the broader context of cutaneous repair, macrophages have been recognized as key inflammatory mediators, although certain observations highlight the significant function of T-cells in modulating the response to inflammation [38]. Evidence has emerged suggesting that MSC-exosomes have the capacity to induce a switch in stimulating T-cells towards a T-regulatory phenotype, thereby mitigating the inflammatory response [39]. Recent research has further unveiled that localized utilization of exosomes can orchestrate the regulation of the interconnected systems of innate and adaptive immunity, culminating in enhanced wound healing [40]. Collectively, these findings underscore the multifaceted impact of MSC-exosomes during the inflammation phase of wound healing. The clinical use of MSCs for skin diseases can potentiate the healing of wounds while mitigating the formation of scars. MSCs travel to the location of cutaneous damage, suppress the inflammatory response, and augment the proliferative and differentiating potentials of endothelial and epidermal cells (Fig. 1) [41].

Taken together, exosomes derived from various MSC sources, including bone marrow, adipose tissue and umbilical cords, have demonstrated therapeutic efficacy in cutaneous wound healing. They achieve this by dimishing inflammation, promoting re-epithelization, stimulating angiogenesis, enhancing fibroblast proliferation and migration, and facilitating ECM formation and remodeling. Nevertheless, the precise mechanisms underpinning exosome-mediated inflammation modulation warrant further elucidation through future investigations. Therefore, the development of technologies aimed at mitigating skin loss due to wounds is imperative.

## Unlocking the potential of MSCs and exosomes in Dermatology: a promising cell-free therapeutic approach

**Fig. 2:**
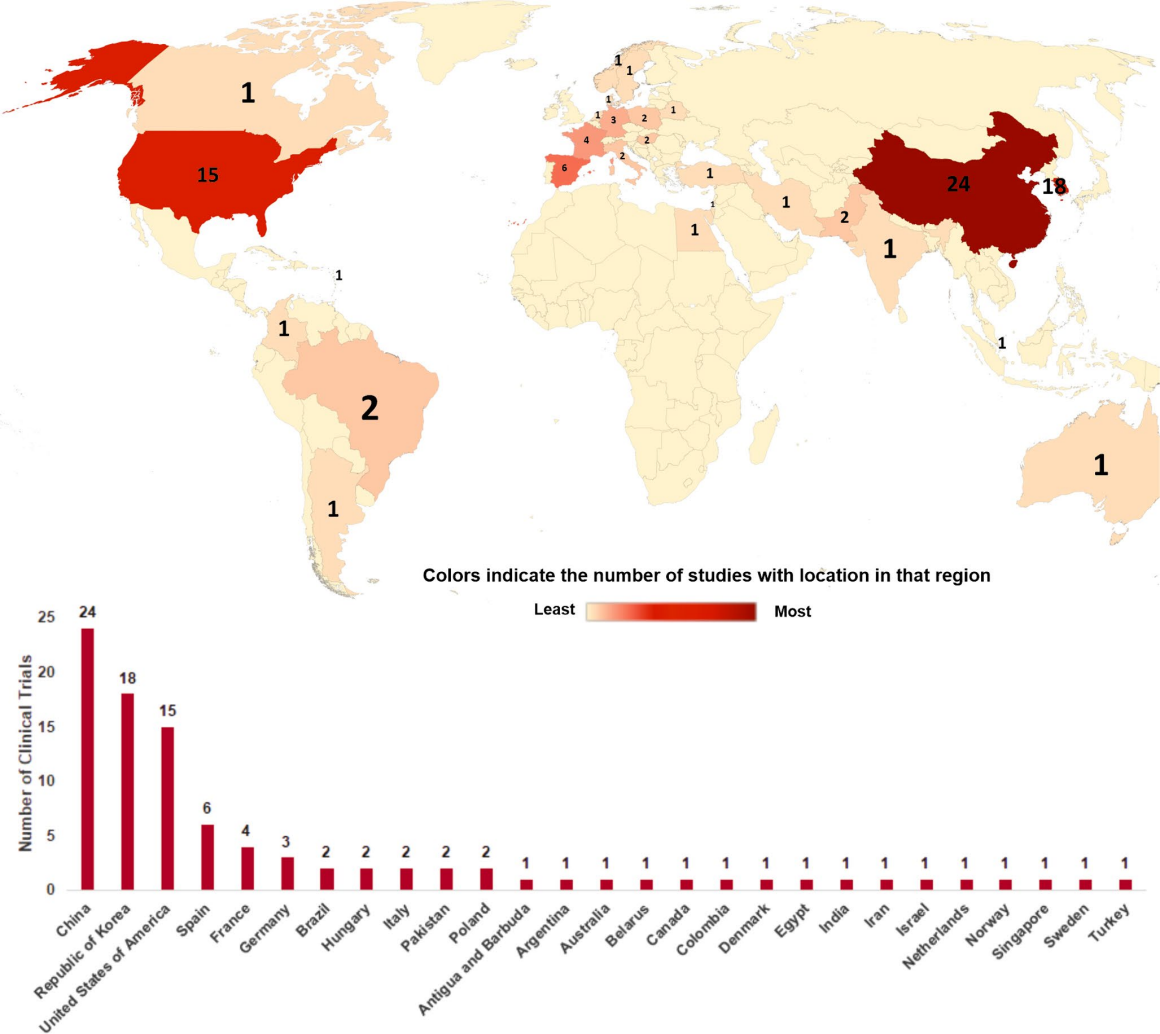
Global distribution of clinical trials based on MSC therapies for accelerating skin diseases listed on ClinicalTrials.gov as of November 2023. The diagram illustrating clinical trials involving MSCs for individuals with skin disorders, categorized by study location. Most of clinical trials were operated in China and Republic of Korea followed by United States of America, Spain, France, Germany while two clinical trials per country were identified in Brazil, Hungary, Italy, Pakistan, and Poland. Countries with only one clinical trial were based in Antigua and Barbuda, Argentina, Australia, Belarus, Canada, Colombia, Denmark, Egypt, India, Iran, Israel, Netherlands, Norway, Singapore, Sweden, and Turkey

**Fig. 3:**
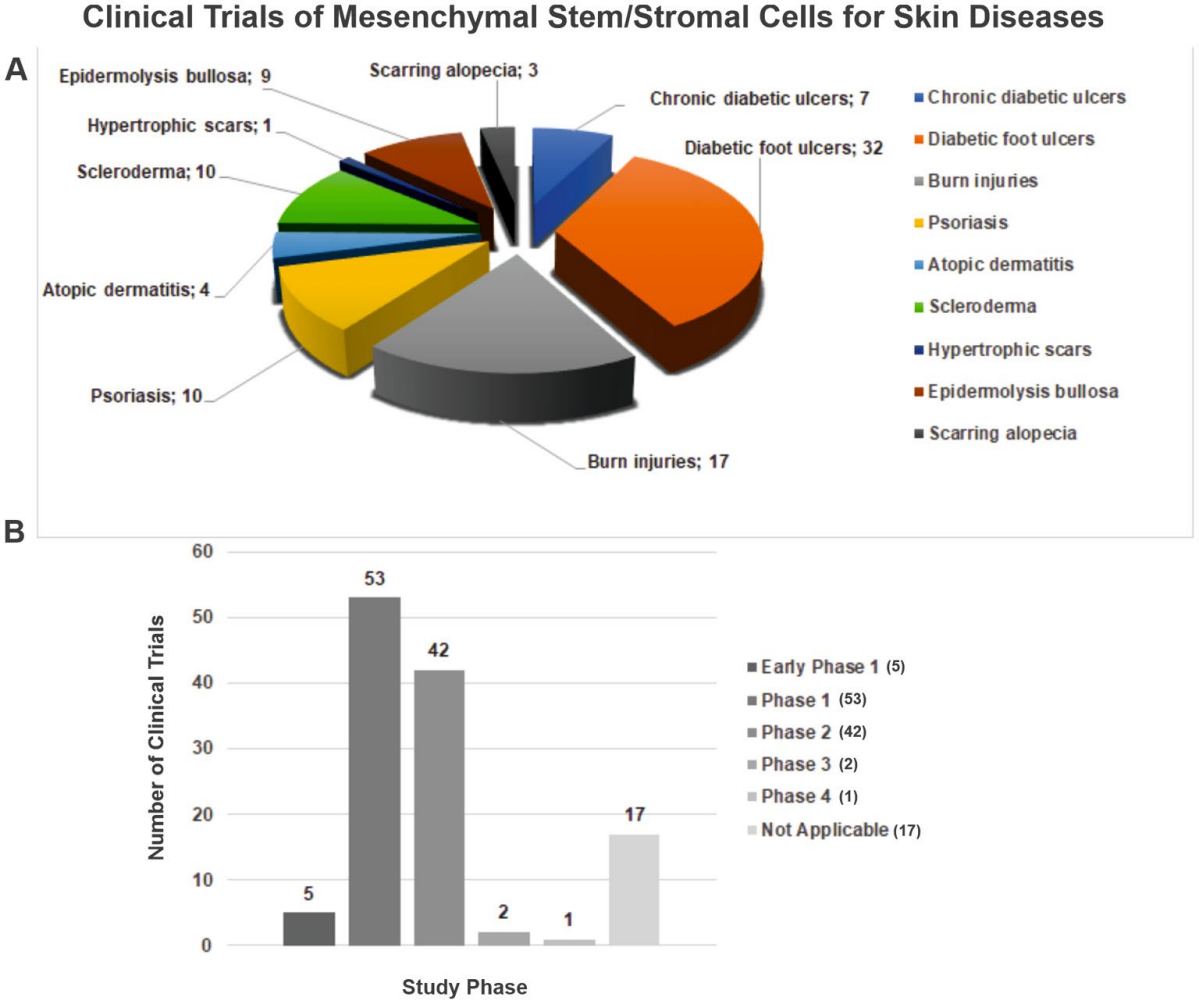
Overview of registered MSC-based clinical trials depending on participants suffering from targeted skin disorders and study phase. (**A**) Pie chart distribution of clinical trials according to sufferers from chronic diabetic ulcers, diabetic foot ulcers, burn injuries, psoriasis, atopic dermatitis, scleroderma, hypertrophic scars, epidermolysis bullosa and scarring alopecia. (**B**) Bar blot of clinical trials related to the investigation phases. Notably, a greater part of clinical trials was in Phase 1 (53) and followed by Phase 2 (42) whereas only one clinical trial was identified in Phase 4. All data was collected by November 14th, 2023

A multitude of investigations have already elucidated the advantageous applications of MSCs in trials, spanning both preclinical and clinical phases. These inquiries have underscored the capabilities of MSCs in the therapeutic management of diverse medical conditions, encompassing blood disorders, diabetes, multiple sclerosis, osteoarthritis, persistent spinal cord injury, and chronic kidney disease in felines [42, 43]. Within the domain of skin health, MSCs have become a consequential modality for augmenting the process of skin wound healing, particularly in the context of immune-mediated pathologies [44]. Furthermore, owing to their immunomodulatory attributes, MSCs have proven efficacious in the treatment of acute graft-versus-host disease that does not respond to steroids [45, 46]. MSCs also hold promise for addressing inflammatory and autoimmune dermatological ailments, including chronic diabetic ulcers [47], diabetic foot ulcers [48], burn injuries [49, 50], psoriasis [51, 52], atopic dermatitis [52, 53], scleroderma [54, 55], hypertrophic scars [56, 57], epidermolysis bullosa [58, 59], vitiligo vulgaris [60] and scarring alopecia [61]. Based on information sourced from the United States National Institutes of Health (NIH) via clinicaltrials.gov, a noteworthy upswing has been observed in the quantity of clinical investigations oriented toward MSCs and exosomes within the preceding year. As of November 2023, a total of 96 trials focused on MSC therapy for skin disorders have been formally recorded in the ClinicalTrials.gov database. Majority of the clinical data were based in China followed by Republic of Korea and the USA, while countries with one trial were Australia, Netherlands, and Turkey (Fig. 2) (http://clinicaltrials.gov). These trials are distributed as follows: 7 trials to chronic diabetic ulcers, 32 trials to diabetic foot ulcers, 17 trials to burn injuries, 10 trials to psoriasis, 4 trials to atopic dermatitis, 10 trials to scleroderma, 1 trial to hypertrophic scars, 9 trials to epidermolysis bullosa and 3 trials to scarring alopecia (http://clinicaltrials.gov, with the data retrieved on 14/11/2023) (Fig. 3).

MSCs possess healing capabilities that render them applicable for the treatment of damaged skin. Their notable characteristics, including ease of acquisition, low immunogenicity, and alignment with natural healing processes, position them as a logical, safe, and efficient therapeutic modality [62]. MSCs exhibit the capacity to migrate to sites of cutaneous injury, quell inflammatory responses, and improve the proliferative and differentiative potential of epidermal cells, fibroblasts, and endothelial cells [41, 63]. Although clinical studies have indicated the safety, feasibility, and effectiveness of interventions based on MSCs [63, 64], it is imperative to acknowledge the limitations inherent in these trials, notably the constrained sample sizes and the absence of extended follow-up data. Notwithstanding certain inconclusive reports, the full validation of MSCs directly differentiating into skin-resident cell phenotypes during cutaneous wound healing remains elusive [65]. These inherent challenges and associated controversies underscore the importance of exploring the therapeutic potential of MSC-secreted factors, particularly exosomes, in the context of healing of cutaneous wounds. Exosomes derived from MSCs, categorized as extracellular vesicles (EVs), encompass a rich cargo of biologically active molecules, including mRNAs, miRNAs, cytokines and growth factors [66, 67]. These exosomes play a pivotal role in promoting re-epithelization, mitigating collagen overproduction, and minimizing scar formation by releasing a plethora of signaling molecules, including transforming growth factor beta (TGF-β), epidermal growth factor (EGF), vascular endothelial growth factor (VEGF), and basic fibroblast growth factor (bFGF) [66, 68]. MSC-derived exosomes have been registered in five clinical trials for skin diseases and aging (clinical trials numbers: NCT05523011, NCT05813379, NCT04173650, NCT05243368 and NCT05658094) and MSC-secretomes including only one clinical trial (clinical trial number: NCT05508191), as demonstrated in Table 1 (https://clinicaltrials.gov/, data retrieved on 14/11/2023). These findings collectively underscore the potential of both MSCs and their derived appendages as candidates to expedite the healing of cutaneous skin diseases. They also provide valuable insights into the intricate biological mechanisms influenced by MSCs and their exosomal constituents, all of which contribute to the enhancement of wound healing and skin regeneration.

**Table 1:** Clinical trials utilizing MSC-derived exosome therapy for skin diseases and injuries

ClinicalTrials.gov Identifier	Conditions	Treatment	Status	Location	Study Type	Phase
NCT05523011	Psoriasis	MSCs-Exosome	Completed	Singapore	Interventional	Phase 1
NCT05813379	Anti-Aging	MSCs-Exosome	Recruiting	Iran	Interventional	Phase 1–2
NCT04173650	Dystrophic Epidermolysis Bullosa	MSCs-Exosome	Not yet Recruiting	United States	Interventional	Phase 1–2
NCT05243368	Diabetic Foot Ulcers	MSCs-Exosome	Recruiting	Spain	Interventional	Not applicable
NCT05658094	Hair Loss, Alopecia	MSCs-Exosome	Recruiting	Iran	Interventional	Not applicable
NCT05508191	Skin Aging, Transepidermal Water Loss	MSC-Secretome	Completed	Indonesia	Interventional	Not applicable

Emerging proof suggests that exosomes originated from MSCs may serve as a viable therapeutic approach without the presence of cells, offering substantial advantages when compared to the use of MSCs themselves. Of particular significance is the reduced risk of tumour formation and lower immunogenicity associated with the administration of MSC-derived exosomes [69]. However, despite these promising attributes, the clinical application of MSC-exosome based therapeutics for skin regeneration encounters certain challenges. These challenges include issues related to suboptimal targeting, swift clearance dynamics, and a comparatively short duration of half-life when operating within the intricate microenvironment of a wound [70]. Additionally, their regulatory status remains a subject of debate. The US Food and Drug Administration (FDA) has yet to officially classify exosomes for the biopharmaceutical industry, leaving the regulatory concerns uncertain [71]. Further development in the quality control of exosome products is essential, indicating a prolonged expedition before their use in clinical practice. Given that exosomes represent a relatively new drug, additional research is required to understand the regulatory consideration of their utilization. Consequently, the subsequent section of this review investigates the underlying mechanisms of MSC-derived exosomes within a 3D platform for cutaneous wound healing and skin regeneration. Such platforms could have the potential to confer enhanced stability and heightened therapeutic efficacy, thereby facilitating the expeditious process of healing wounds and regenerating skin.

## Enhancing the therapeutic efficacy of MSC-exosomes through biomaterial synergy: a 3D approach to skin regeneration

Notwithstanding the extensive evidence presented in the former sections, underscoring the favourable impact of MSCs and their exosomes on the process of cutaneous wound healing and skin regeneration in animal models and preclinical trials [72, 73], it is worth noting that the clinical data regarding the application of exosomes derived from MSCs in the treatment of cutaneous wounds remains limited. As these MSC-exosomes are poised for translation into clinical practice, there is a pressing need to enhance their therapeutic efficacy. The overarching objective of this research is to amplify the capacity of MSC-exosomes in expediting the wound healing process. A potential solution could be combining exosomes with biomaterials to synergistically improve their functions. Specifically, exosomes within hydrogels can be sustainably released over a long period of time, contributing to lasting therapeutic effects. Tailoring biomaterials offers a more versatile platform for exosome approaches. In comparison to the delivery of cells, the incorporation of MSCs within a biomaterial matrix is able to boost their wound healing capabilities by strategically placing the cells at the site of the defect and the concurrent upregulation of trophic factor secretion including EGF, TGFB, VEGF, bFGF. Beyond the effects on trophic factors, it has been observed that MSC-seeded scaffolds can upregulate the expression of matrix metalloproteinase 9 (MM9) within the ECM, while also augmenting the recruitment of endogenous progenitor cells during the course of tissue repair [74]. Furthermore, investigations have demonstrated that adipose tissue derived MSCs, when embedded within scaffolds, exhibit the capacity to suppress TNFα-dependent inflammation, amplify the population of M2 macrophages, stimulate angiogenesis through TGF-β1-mediation, enhance myoblast differentiation, and facilitate the establishment of granulation tissue [74].

Optimal biomaterials serve as indispensable vehicles to potentiate the therapeutic attributes of MSC-exosomes when employed as wound dressings, imparting heightened endurance and steadiness to these bioactive entities. A diverse array of materials in biomaterial category, including nanofibers produced by electrospinning, nanoparticles in colloidal form, hydrogels and membranes, have been harnessed to enable the regulation of active biological agents in the context of skin regeneration [75]. Among the gamut of biomaterials serving as hosts for exosome delivery, hydrogels have emerged as particularly user-friendly, cost-effective, and accessible options. Consequently, hydrogels have garnered extensive utilization as a vehicle for the continuous and effective administration of exosomes [76]. Notably, hydrogels have garnered recent attention for novel cell-free approaches as scaffold dressings, endowing them with versatile characteristics, encompassing hemostatic capability, antibacterial efficacy, anti-ultraviolet activity, tissue adherence, injectability, and self-healing attributes [77]. Hydrogels function as three-dimensional (3D) porous frameworks that mimic the architecture of the natural ECM, thereby furnishing a 3D structure that supports the growth of encapsulated cells, while simultaneously orchestrating the activation of biomolecules [78]. Furthermore, 3D water-attracting polymer matrices effectively retain moisture in the area of damaged tissues. The amalgamation of hydrogels with MSC-exosomes assumes a pivotal role in modulating the wound’s inflammatory microenvironment, attenuating collagen deposition, fostering vascularization, expediting re-epithelialization, and accelerating the overall wound healing process [79]. A composite comprising hydrogels with exosomes can also function as a sustained-release system, exerting long-lasting therapeutic effects, thus engendering heightened research interest [80]. MSC-exosomes encapsulated within biomaterials exhibit the advantage of evading rapid release into the circulatory system and instead execute their remedial actions in a manner dependent on the dosage at predetermined locations.

In vivo investigations have demonstrated that the incorporation of exosomes originated from gingival MSCs into a hydrogel based on chitosan and silk can significantly enhance the healing processes of cutaneous wounds. This enhancement is evidenced by the promotion of new blood vessels, the restoration of epithelial tissue, and the rearrangement of collagen. Previous research has also indicated the potential of hydrogel containing acrylic acid in promising skin regeneration, as validated in murine injuries from burns [81]. Moreover, a hydrogel based on chitosan, enriched with exosomes from MSCs, has exhibited the capacity to augment the proliferation and migration of fibroblasts in vitro, along with stimulating re-epithelialization in vivo [82]. In research conducted by Wang and colleagues, a hydrogel based on injectable polypeptides, laden with exosomes originated from adipose tissue MSCs, was developed to synergistically enhance the efficiency of healing diabetic cutaneous wounds. This construct facilitated the regeneration of epithelial tissue and the accumulation of collagen at the injury site [83]. In a recent study, the integration of exosomes originated from gingival MSCs into gel-like substances comprised of chitosan and silk efficiently aided in the recovery of diabetic skin wounds by promoting re-epithelialization, collagen accumulation, angiogenesis, and neuronal ingrowth [84]. Spheroids of MSCs encapsulated in more rigid gels displayed the elevated expression of the anti-inflammatory factor PGE2 and yhe pro-angiogenic factor VEGF in comparison with the untreated MSCs. This phenomenon stimulated the proliferation of endothelial cells, improved the polarization of macrophages, and accelerated angiogenesis in vivo [85]. Similarly, Wang and colleagues exhibited the use of exosomes composed of hydrogels, as biocompatible natural materials such as methylcellulose and chitosan in experimental models of severe wounds under diabetic conditions [86]. More recently, in rat models, the utilization of a genipin crosslinked hydrogel loaded with exosomes derived from UC-MSCs facilitated the recovery of skin wounds extending through all layers. These exosomes notably enhanced the sealing of wounds, the speed of epithelial tissue regeneration, and reinforced the deposition of collagen in the wound tissue [87]. The modified exosome hydrogel formulation provided as an injectable wound dressing, exhibiting suitable time for gelation, mechanical characteristics, and remarkable self-repair abilities. In a model of diabetic wound infection, exosomes derived from MSCs in the bone marrow were incorporated into an antibacterial hydrogel. This formulation effectively adjusted the inflammatory microenvironment of the wound, fostered neovascularization, and accelerated the process of recovery [79]. An innovative study by Hu et al. reported cryogenic 3D printing using extrusion-based technology to fabricate a 3D scaffold dressing (SIS/MBG@Exos) by combining decellularized small intestinal submucosa (SIS) integrated with mesoporous bioactive glass (MBG) and exosomes. This scaffold enabled the sustained release of exosomes [88]. In vitro experiment, the combined hydrogel scaffolds were shown to enhance the proliferation, migration, and angiogenesis of human umbilical vein endothelial cells (HUVECs), while in vivo experiment, they assisted to improve blood flow and to trigger the angiogenesis process in diabetic wounds, thereby promoting diabetic wound healing. Additionally, these engineered scaffolds encouraged the development of granulation tissue, the placement of collagen fibers, and the progress of new blood vessels [88].

Collectively, the above-mentioned strategies serve to enhance the therapeutic efficacy of exosomes in the context of cutaneous wound healing and skin regeneration. Despite promising prospects, it is crucial to realize that clinical application of exosomes encounters significant obstacles related to safety concerns and quality controls. The process of translating MSC-exosomes into clinical practice lacks a predictable solution. Consequently, the practical application of exosomes in clinical settings needs further research depending on stability and overcoming these challenges.

## Prospects and conclusion

In light of the growing demand for efficient therapeutic interventions to facilitate skin regeneration, a persistent challenge in public health, the need for innovative solutions becomes increasingly pronounced. This challenge is expected to escalate alongside the rising prevalence of long-term disease conditions and the overall aging of the populace, leading to an extended average life expectancy. As a prospective treatment strategy, therapies utilizing exosomes have emerged as an encouraging approach for the promotion of wound healing. The comprehensive examination conducted in this review highlights the considerable promise of exosomes as potential therapeutic interventions, particularly in addressing persistent non-healing wounds. Additionally, exosomes have exhibited the potential to mitigate the development of scars, alleviate inflammatory responses at the affected site, enhance the formation of granulation tissue, and induce the growth of fibroblasts in the dermis. The cumulative findings emphasize the healing efficacy of exosomes in facilitating the recovery process of wounds. In recent years, significant strides have been made in integrating exosomes with inventive engineering approaches, further amplifying their utility as valuable tools for wound healing. Noteworthy advantages associated with exosome-based therapies include their abundant sources, straightforward preparation, convenient storage and transport, and minimal likelihood of triggering immune responses.

Notwithstanding the considerable potential of exosomes in the treatment of wounds, several challenges remain. It is crucial to conduct extensive research to enhance our comprehension of exosome biology, ensuring the secure and efficient application of these therapies. Substantial additional investigation efforts are essential to expedite the accessibility for purchase and the utilization in clinical settings for exosome-based treatments. The significant diversity observed in biomaterials, cell sources, and administration routes currently being explored underscores the pressing requirement for additional investigation in this realm. Notably, the dearth of such relative studies represents a significant void that necessitates immediate attention. The extension of follow-up periods is crucial to comprehensively assess the influence of exosomes on the recovery process of skin wounds, along with their impact on the elicited immunological response. Furthermore, forthcoming research endeavours should encompass a broader spectrum of skin lesions, including incisional ischemic lesions, ulcers and burn injuries, while utilizing more accurate representations of type 2 diabetes and wounds that do not heal.

In summary, the outcomes of the research outlined in this review substantiate the evidence that exosomes possess remarkable potential for therapeutic interventions in the healing process of wounds. Multidisciplinary research and collaborative efforts are essential to respond to scientific inquiries and overcome technical challenges in the utilization of exosomes, thus closing the disparity between experimental investigations and the commercialization process. As advances in exosome research continue to emerge and there are persistent efforts to overcome these challenges, therapies involving exosomes may become important clinical therapies. Achieving consensus on variables in this field could accelerate progress and be a promising treatment that will seamlessly translate exosome-based therapies into clinical practice.

## Data Availability

No datasets were generated or analysed during the current study.
